# Type 1 diabetes mellitus in children: Patient reported outcomes

**DOI:** 10.1371/journal.pone.0322882

**Published:** 2025-05-05

**Authors:** Basima A. Almomani, Roa’a N. Elayyan, Samah F. Al-Shatnawi

**Affiliations:** Department of Clinical Pharmacy, Faculty of Pharmacy, Jordan University of Science and Technology, Irbid, Jordan; Sohag University Faculty of Medicine, EGYPT

## Abstract

The global prevalence of type 1 diabetes mellitus (T1DM) is increasing. Poor glycemic control in children and adolescents leads to both acute and chronic problems, reduced Health Related Quality of Life (HRQoL), and higher healthcare utilization. This study aimed to assess patient-reported outcomes related to self-management adherence, QoL, diabetes-related stigma, glycemic control, and other clinical outcomes along with their determinants, in insulin-treated pediatric T1DM patients in Jordan. A cross-sectional study was conducted from April to October 2023 at two health centers in Northern Jordan. Eligible pediatric T1DM patients attending outpatient clinics were enrolled. Trained pharmacists conducted face-to-face interviews with both children and their guardians, using validated tools that were translated into Arabic. Adherence was evaluated using the Diabetes Management Questionnaire, HRQoL was measured using the Pediatric Quality of Life Inventory 3.0 Diabetes Module, stigma was assessed using the Child Attitude Toward Illness Scale and glycemic control was determined by glycated hemoglobin levels. A total of 150 patients participated in the study. The mean adherence score was 57.4 ± 18.13. Factors such as younger age (P-value = 0.01), higher monthly income (P-value = 0.022) and shorter disease duration (P-value = 0.008) were associated with improved adherence. The mean pediatric QoL score was 63.27 ± 11.86, with male gender (P-value = 0.021) and the absence of disease-related factors (P-value = 0.004) linked to lower QoL scores. Additionally, body mass index (P-value = 0.041) and a family history of DM (P-value = 0.047) were linked to stigma. Most patients (76%) had uncontrolled diabetes, with disease duration (P-value = 0.019) and maternal educational level (P-value = 0.013) influencing glycemic control. These findings highlight that, despite widespread poor glycemic control, insulin adherence and QoL among pediatric T1DM patients in Jordan are above average. Targeted interventions are recommended to improve adherence and, in turn, overall patient outcomes.

## Introduction

The prevalence of diabetes mellitus (DM) is rising globally, with Middle East and North Africa (MENA) region reporting the highest rate of DM worldwide [1,2]. This trend places significant strain on the global economy, with DM-related expenditures totaling a staggering 966 billion dollars [3]. Diabetes mellitus type 1 (T1DM) is primarily caused by the autoimmune destruction of beta cells in the pancreas, resulting in insufficient insulin production [4]. According to International Diabetes Federation (IDF), nearly 1.2 million children and adolescents were living with T1DM in 2021 [5].

Adherence to T1DM management is crucial for disease effective control and is closely linked to factors such as family support, knowledge of DM, and the duration of the disease [6]. Poor adherence to T1DM management- encompassing insulin use, nutritional guidelines, and physical activity- has been shown to negatively impact glycemic control [7]. Consequently, glycemic control, as measured by HbA1c levels, is strongly associated with the occurrence of diabetic ketoacidosis (DKA), hypoglycemia, emergency department visits, and overall health-related quality of life (HRQoL) [8–11]. Additionally, both DKA and hypoglycemia, two major acute complications of DM, have been found to be linked with younger age and inadequate diabetes education [10]. Ongoing education and awareness of DM and its management are key in improving patients’ overall health. The stigma surrounding T1DM presents a significant barrier to adherence, thus severely impacting glycemic control [12]. Moreover, social rejection and lack of concern regarding DM have been shown to lead to the development of additional complications [13]. Quality of life (QoL) is a critical outcome and an important focus of healthcare intervention. Episodes of hypoglycemia and frequent hospitalization are associated with a lower QoL [14]. Additionally, children who experience higher school absenteeism often report poorer QoL, particularly in terms of their school functioning [13].

In Jordan, research by Alassaf et al. revealed that a majority of children and adolescents with T1DM (79.1%) had poor glycemic control, with age, dietary adherence, and insulin administration at school identified as predictors of glycemic control [15]. Furthermore, a significant proportion of individuals with T1DM experienced hypoglycemia in the previous 6 months, with many being aware of the symptoms of hypoglycemia. Notably, 43% of patients had experienced episodes of DKA [16,17]. According to Alkhatatbeh et al., frequent episodes were linked to lower levels of hypoglycemia awareness [16]. Additionally, Aljawarneh et al. found that adolescent T1DM patients reported lower HRQoL and modest adherence to insulin therapy [18]. However, there is limited information on HRQoL, medication adherence, diabetes-related stigma, and complications in pediatric T1DM patients. This study aims to assess patient-reported outcomes among insulin-treated pediatric patients with T1DM in Jordan. Specifically, it evaluates self-management adherence, quality of life, diabetes-related stigma, glycemic control and acute diabetes-related complications (e.g., hypoglycemia, DKA). Additionally, the study examines sociodemographic and clinical factors influencing these outcomes to provide insights that can inform targeted interventions for improving pediatric diabetes management.

## Subjects, materials and methods

### Study design and patient recruitment

A cross-sectional study was conducted involving pediatric patients diagnosed with Type 1 Diabetes Mellitus (T1DM) at two leading referral teaching hospitals in Northern Jordan: King Abdullah University Hospital (KAUH) and Princess Rahma Teaching Hospital (PRTH) between April 25, 2023, and October 22, 2023. The sample size was calculated using the Roasoft online calculator, with a margin of error of 5%, a population size of 1300 based on data from the International Diabetes Federation (IDF) [19], a 50% response rate, and an 80% confidence level, resulting in a target sample of 146 participants. For context, previous cross-sectional studies conducted in Jordan by Sabbah et al. and Alkatatbeh et al. included sample sizes of 109 and 94 pediatric T1DM participants, respectively [16,20].

The lead researcher, a clinical pharmacist, engaged eligible pediatric patients and their guardians at the pediatric endocrine clinics of the participating institutions, providing invitations and explaining the study’s objectives. The inclusion criteria consisted of children under 18 years of age who had a confirmed diagnosis of T1DM by an endocrinologist for at least six months and were using multiple daily injection (MDI) insulin therapy. Patients who were newly diagnosed, or had immunocompromised conditions, cognitive impairments, or developmental issues were excluded from the study. The study was conducted in the outpatient clinic’s waiting area, where face-to-face interviews with both children and their guardians were facilitated by a trained clinical pharmacist. Before starting the interviews, the researcher received thorough training through regular meetings with the research team to ensure consistent and accurate administration of questions. During the interviews, the researcher presented study questions in a clear and straightforward manner, addressing any ambiguity with simplified language and visual aids such as insulin pictures to aid understanding. On average, interviews lasted between 15–20 minutes per participant, whether with the guardian or the child. Generally, children aged 12 years or older completed the questionnaire themselves, while those younger than 12 had the guardian complete it.

Ethical considerations were a top priority throughout the study. Approval was obtained from the institutional review boards (IRB) at Jordan University of Science and Technology (15/158/2023) and the Ministry of Health (MOH/REC/2023/159) before the research commenced. Written informed consent was obtained from all guardians, while children aged 12 years and older provided assent prior to participating in the study. This process ensured compliance with ethical standards and upheld the principles of respect for autonomy, beneficence, and justice in research involving human subjects.

### Outcome measures

Adherence was assessed using the 20-item Diabetes Management Questionnaire (DMQ) developed by Mehta et al. [21], a reliable and validated tool designed to evaluate diabetes self-management adherence among pediatric T1DM patients aged 8–18 years. The questionnaire covers four key areas: insulin delivery, blood glucose monitoring, dietary habits, and physical activity. Since an Arabic version of the tool was unavailable, a forward-backward translation process was conducted, demonstrating high consistency between the original and translated versions. The internal consistency of the questionnaire in our study, measured using Cronbach’s alpha, was 0.842. Each item includes response options ranging from “0 = never” to “4 = almost always,” with six out of 20 items reversed. The mean score of the items was calculated, multiplied by 25, and normalized to a 0–100 scale, with higher scores indicating better adherence [21].

Health-related quality of life (HRQoL) was measured using the validated Arabic version of the Pediatric Quality of Life Inventory 3.0 Diabetes Module [22], originally developed by Varni et al. [23]. This 28-item tool evaluates the HRQoL perceptions of both T1DM patients and their guardians. In our study, Cronbach’s alpha for HRQoL report was 0.747. Two versions of the questionnaire were available: a child report for those aged 12 and older, and a parent proxy report. The questionnaire includes five domains: symptoms (11 items), treatment barriers (4 items), treatment adherence (7 items), worry (3 items), and communication (3 items). Responses are rated on a 5-point Likert scale, ranging from 0 (never) to 4 (almost always). Items scores were reversed and transformed into a 0–100 scale, with higher scores indicating better HRQoL [23].

Stigma was assessed using the Child Attitude Toward Illness Scale (CATIS) [24], a validated 13-item tool developed by Austin and Huberty for participants aged 8–22 years. The Arabic version of this tool was previously used in a study among pediatric asthma patients in Jordan [25]. In our study, the internal consistency, measured by Cronbach’s alpha, was 0.814. Responses were rated on a 5-point scale, ranging from 1 to 5 (with 1 indicating strongly negative feelings and 5 indicating strongly positive feelings). The sum of the 13 items scores was averaged, with higher scores indicating a lower perception of stigma [24].

Additional information collected included participants’ awareness of hypoglycemia and diabetic ketoacidosis (DKA), as well as demographic and clinical characteristics such as age, gender, height, weight, residence, mother’s education level, family history of DM, family monthly income, age at diagnosis, disease duration, glycated hemoglobin (HbA1c %), blood glucose level, comorbidities, information on hypoglycemia and DKA episodes, and disease-related factors (frequency of school absences, emergency department visits, or hospital admissions) within the previous 6 months.

### Statistical analysis

Statistical analysis was performed using IBM SPSS version 22 (Armonk, NY; IBM corporation). Continuous variables were presented as mean ± standard deviation (SD) or median [interquartile range (IQR)], while categorical variables were expressed as frequencies and percentages. Parametric or non-parametric tests were applied as appropriate. Factors associated with adherence, HRQoL, and stigma were evaluated using independent t-tests and correlation analyses. The relationship between glycemic control, DKA occurrence, and various factors was assessed using independent t-tests, Chi-square tests, and Mann-Whitney tests. Additionally, factors related to hypoglycemia occurrence were analyzed using Mann-Whitney tests and Spearman’s correlation. Normality of the data was assessed using histograms and quantile-quantile (Q-Q) plots, with normality assumed if the distribution was symmetrical and the Kolmogorov-Smirnov and Shapiro-Wilk tests yielded P-values > 0.05. Levene’s test was used to assess the homogeneity of variance, with a P-value > 0.05 indicating equal variance across groups

All variables with P-values < 0.1 in univariate analyses were included in multivariate analyses, which employed linear or binary logistic regression models. The normality of residuals was assessed using a histogram, with a symmetrical distribution indicating normality. Logarithmic transformation was applied to hypoglycemia-associated factors when normality was not achieved.

Homoscedasticity and linearity were assessed using scatterplots of residuals versus predicted values; a symmetrical distribution around the horizontal line indicated linearity and constant variance. Multicollinearity was evaluated using the Variance Inflation Factor (VIF), with values < 5 suggesting the absence of multicollinearity. The Durbin-Watson statistic was used to assess the independence of residuals, with values between 1 and 3 indicating independence. Influential or outlier points were identified using Cook’s distance, with values < 1 suggesting no significant outliers. A significance level of P < 0.05 was considered statistically significant for all analyses.

## Results

### Demographic and clinical characteristics

Out of the 155 guardians and their children approached for the study, 150 were included. The mean age of the children was 10.46 ± 3.3 years, with an equal distribution between genders, with 50% being females. Regarding maternal education, less than half (39.3%) of the mothers had attained a university degree, while the majority (59.3%) reported a monthly family income of ≥ 500 JD. Notably, approximately 60% of the children had a family history of DM, and 16.7% had comorbid conditions. The mean age at diagnosis was 7.27 ± 3.61 years, with a median duration of DM of 3 years. However, only a quarter of the children (24%) had controlled DM (HbA1c < 7%), and roughly half (48%) had a blood glucose level of ≤ 130 mg/dL. These findings are summarized in Table 1.

**Table 1:** Demographic, clinical and outcomes characteristics of study participants.

Characteristics^a^	Values
Age (years)^b^	10.46 ± 3.3
Participants age groups	
< 12 years old	88 (58.7%)
≥ 12 years old	62 (41.3%)
Body Mass Index (Kg/m^2^)^b^	18.66 ± 3.59
Mother’s level of education	
Basic education	91 (60.7%)
University education	59 (39.3)
Place of Residence	
Urban areas	107 (71.3%)
Rural areas	43(28.7%)
Family’s monthly income	
< 500 JD	61 (40.7%)
≥ 500 JD	89 (59.3%)
History of DM in family	
No	61 (40.7%)
Yes	89 (59.3%)
Age at diagnosis^b^	7.27 ± 3.61
DM duration (years)^c^	3 (1-5)
HbA1c level categories^d^	
< 7% [53 mmol/mol] (controlled)	36 (24%)
≥ 7% [53 mmol/mol] (uncontrolled)	114 (76%)
Fasting blood glucose level categories^d^	
≤ 130 mg/dl	72 (48%)
> 130 mg/dl	78 (52%)
Comorbidities	
No	125 (83.3%)
Yes	25 (16.7%)
Disease related factors^e^	
No	59 (39.3%)
Yes	91 (60.7%)
**Diabetes Management Questionnaire (DMQ) categories^f^**	
Adherence to physical activity	25.39 ± 34.41
Adherence to diet management	54.56 ± 22.91
Adherence to glycemic monitoring	64.64 ± 18.58
Adherence to insulin use	82.5 ± 19.87
Total DMQ Score	57.4 ± 18.13
**Pediatric Quality of Life (PedsQL) 3.0 version aspects^f^**	
Diabetes symptoms	59.5 ± 15.08
Treatment barriers	60.5 ± 24.4
Treatment adherence	71.09 ± 15.07
Worry	46.67 ± 24.33
Communication	79.11 ± 31.96
Total score	63.27 ± 11.86

Abbreviations: JD, Jordanian Dinar; DM, Diabetes Mellitus.

^a^All data was expressed as n (%) of participants unless otherwise indicated.

^b^Data were described as mean ±SD - (normal distribution).

^c^Data were described as median [Interquartile range] – (Skewed distribution).

^d^HbA1c and fasting blood glucose level were classified according to American Diabetes Association (ADA) guidelines.

^e^Include school absent, emergency room visits and hospital admission.

^f^Data was described as correlation coefficient and analyzed by Pearson correlation.

### Outcome measures

The overall mean adherence score among participants was 57.4 ± 18.13 out of 100, with the highest adherence observed in insulin use (82.5 ± 19.87) and the lowest in physical activity (25.39 ± 34.41). A comprehensive breakdown of the Diabetes Management Questionnaire (DMQ) domains is presented in Table 1. Multivariate analysis (Table 2) revealed that higher adherence score was associated with younger age (β = -8.404; P-value = 0.01), higher family monthly income (β = 6.843; P-value = 0.022), and shorter duration of diabetes (β = -1.406; P-value = 0.008). The mean HRQoL score was 63.27 ± 11.86 out of 100. Participants scored highest in the communication domain (79.11 ± 31.96) and lowest in the worry domain (46.67 ± 24.33), as shown in Table 1. Additionally, male patients had significantly higher HRQoL scores (β = 4.133; P-value = 0.021), while the presence of disease-related factors was associated with lower HRQoL scores (β = -5.421; P-value = 0.004), as shown in Table 3.

**Table 2:** Univariate and multivariate analyses of factors associated with adherence to DM management.

Characteristics	Univariate analysis		Multivariate analysis^e^	
	N = 150		N = 150	
	Participants	P-value	β	P-value
Gender^a^		0.893		
Male	57.2 ± 19.28			
Female	57.6 ± 17.03			
Patients’ age group^a^		<0.001	- 8.404	0.01
< 12 years	61.68 ± 16.9			
≥ 12 years	51.33 ± 18.22			
Patients’ age group^a^		<0.001	-7.739	0.007
< 12 years	61.68 ± 16.9			
≥ 12 years	51.33 ± 18.22			
BMI^b^	-0.136	0.097	0.19	0.672
Mother’s level of education^a^		0.009	4.463	0.158
Basic education	54.29 ± 18.25			
University education	62.20 ± 17.01			
Family’s monthly income^a^		0.003	6.843	0.022
< 500 JD	52.09 ± 17.99			
≥ 500 JD	61.04 ± 17.41			
Residence^a^		0.054	- 4.667	0.138
Urban areas	59.21 ± 18.77			
Rural areas	52.91 ± 15.75			
Family history for DM^a^		0.649		
No	58.22 ± 17.72			
Yes	56.84 ± 18.49			
Age at diagnosis (years)^b^	-0.134	0.103		
Duration of diabetes (years)^c^	-0.262	0.001	- 1.406	0.008
HbA1c^a^		0.021	- 2.499	0.469
< 7%	63.47 ± 19.01			
≥ 7%	55.48 ± 17.5			
Blood glucose level^a^		0.175		
≤ 130 mg-dl	59.5 ± 19.1			
> 130 mg/dl	55.46 ± 17.13			
Comorbidities^a^		0.306		
No	58.08 ± 18.2			
Yes	54 ± 17.78			
Number of hypoglycemia episodes in the previous 6 months^c^	0.126	0.125		
DKA occurrence in the previous 6 months^a^		0.449		
No	58.1 ± 18.58			
Yes	55.59 ± 17.01			
Disease related factors^a^^,^^d^		0.929		
No	57.56 ± 18.34			
Yes	57.29 ± 18.1			
Stigma score^b^	-0.027	0.744		
HRQoL score^b^	0.169	0.039	0.076	0.535

Abbreviations: BMI, Body Mass Index; HbA1c, Glycated hemoglobin; HRQoL, Health Related-Quality of Life; DKA, Diabetic Ketoacidosis; JD, Jordanian Dinar.

^a^Data was described as mean ±SD and analyzed by independent t-test.

^b^Data was described as correlation coefficient and analyzed by Pearson correlation.

^c^Data was described as correlation coefficient and analyzed by Spearman correlation.

^d^Include school absent, emergency room visits and hospital admission.

^e^Multivariate analysis: linear regression.

**Table 3:** Univariate and multivariate analyses of factors associated with HRQoL.

Characteristics	Univariate analysis		Multivariate analysis^e^	
	N = 150		N = 150	
	Participants	P-value	β	P-value
Gender^a^		0.01	4.133	0.021
Male	65.75 ± 10.79			
Female	60.79 ± 12.43			
Patient’s age group^a^		0.448		
< 12 years	63.93 ± 9.64			
≥ 12 years	62.33 ± 14.48			
BMI^b^	-0.031	0.708		
Mother’s level of education^a^		0.002	3.584	0.072
Basic education	60.86 ± 12.56			
University education	66.98 ± 9.67			
Family’s monthly income^a^		0.003	2.426	0.22
< 500 JD	59.86 ± 13.27			
≥ 500 JD	65.6 ± 10.23			
Residence^a^		0.128		
Urban areas	64.2 ± 12.08			
Rural areas	60.94 ± 11.09			
Family history for DM^a^		0.36		
No	62.19 ± 12.72			
Yes	64 ± 11.25			
Age at diagnosis (years)^b^	-0.07	0.423		
Duration of diabetes (years)^b^	-0.013	0.874		
HbA1c^a^		0.008	- 3.018	0.166
< 7%	67.81 ± 12.79			
≥ 7%	61.83 ± 11.23			
Blood glucose level^a^		0.033	- 2.773	0.125
≤ 130 mg/dl	65.41 ± 10.31			
> 130 mg/dl	61.29 ± 12.88			
Comorbidities^a^		0.227		
No	63.79 ± 11.57			
Yes	60.64 ± 13.16			
Number of hypoglycemia episodes in the previous 6 months^c^	-0.078	0.346		
DKA occurrence in the previous 6 months^a^		0.01		
No	64.81 ± 11.37			
Yes	59.29 ± 12.31			
Disease related factors^a^^,^^d^		0.001	- 5.421	0.004
No	67.33 ± 10.81			
Yes	60.64 ± 11.82			
Adherence score^b^	0.169	0.039	0.052	0.316
Stigma score^b^	-0.018	0.826		

Abbreviations: BMI, Body Mass Index; HbA1c, Glycated hemoglobin; HRQoL, Health Related-Quality of Life; DKA, Diabetic Ketoacidosis; JD, Jordanian Dinar.

^a^Data was described as mean ±SD and analyzed by independent t-test.

^b^Data was described as correlation coefficient and analyzed by Pearson correlation.

^c^Data was described as correlation coefficient and analyzed by Spearman correlation.

^d^Include school absent, emergency room visits and hospital admission.

^e^Multivariate analysis: linear regression.

The mean stigma score among our participants was 2.64 ± 0.33 (out of 5). As shown in S1 Table, patients with lower BMI levels (β= - 0.016; P-value = 0.041) and those without a family history of DM exhibited higher stigma scores compared to others (β= - 0.109; P-value = 0.047). A significant majority of our patients (76%) had uncontrolled disease (HbA1c ≥ 7%), and multivariate analysis (S2 Table) revealed that children with shorter disease durations (OR = 1.272; 95%CI [1.040–1.557]; P-value = 0.019) and those whose mothers had a university education (OR = 0.325; 95%CI [0.133–0.793]; P-value = 0.013) had lower HbA1c levels, indicating better glycemic control.

In the previous 6 months, the majority of patients (94%) experienced at least one episode of hypoglycemia and used various carbohydrate sources, such as juice, dates, sugar with water, and honey to relief the condition. Among the reported symptoms, shaking was the most common autonomic symptom, while dizziness was the most frequently reported neuroglycopenic symptom. Additionally, more than a quarter of patients (28%) experienced episodes of DKA during the same period. Features of hypoglycemia and DKA episodes among our patients are summarized in S3 Table. Lower BMI was associated with a higher occurrence of hypoglycemia (β= - 0.084, P-value = 0.003) (S4 Table). In contrast, a lower HRQoL score was significantly associated with DKA occurrence (OR = 0.962; 95%CI 0.933–0.992; P-value = 0.012), as indicated in S5 Table.

## Discussion

The present study provides insights into the patients-reported outcomes and their determinants among Jordanian pediatric patients withT1DM. Three key findings emerged from our investigation. First, our patients demonstrated better adherence to insulin use than to dietary and physical activity recommendations. Second, they reported higher-than-average HRQoL and moderate levels of stigma. Third, the majority of patients struggled with uncontrolled diabetes and experienced hypoglycemia episodes in the past 6 months.

Adherence to diabetes management is essential for optimizing glycemic control, reducing hospital admissions and emergency room visits, and easing the burden on healthcare resources [26]. Our study found an adherence score of 57.4, which is lower than that reported in previous studies using the same assessment tool in Jordan [20] and other countries [27,28]. This discrepancy may be attributed to differences in the proportions of educated mothers, as earlier research predominantly focused on industrialized countries with better access to healthcare. Notably, our participants demonstrated the highest adherence to insulin use and the lowest adherence to physical activity, which aligns with findings by Rochmah et al. [7]. Despite the essential role of insulin administration in Jordan’s healthcare system, children often spend their free time engaged in sedentary activities, such as watching TV and playing video games, which limits opportunities for outdoor play and physical activity. To improve adherence and disease management, it is crucial to strengthen the roles of clinical pharmacists and dietitians in patient education and promote the use of adherence tools, including telemedicine. These interventions can support regular monitoring, provides personalized guidance, and empower patients to follow their treatment regimens more effectively. By fostering a collaborative healthcare approach and leveraging technological advancements, we can work towards better diabetes management and improved outcomes for pediatric T1DM patients.

In our current study, we found that younger children exhibited better adherence compared to older children, a result consistent with Sabbah et al. [20]. However, in contrast to an earlier study, we observed that a longer duration of DM was associated with lower adherence scores [7]. At the time of diagnosis, parents typically play a significant role in disease management, but as patients transition into adolescence, parental involvement often decreases, which can leading to potential conflicts and subsequently, poorer treatment adherence [29]. Additionally, our study revealed a positive association between higher family income and adherence scores, which contradicts the findings of Yeo et al. [30].

The Health-Related Quality of Life (HRQoL) among our participants was reported at 63, slightly lower than the score reported by Rochmah et al. [7]. We found that participants scored highest in the communication domain and lowest in the worry domain, which is consistent with the findings of Cho et al. [31]. Furthermore, our study revealed that female patients and those with disease-related factors experienced lower HRQoL, aligning with previous research [32,33]. These findings highlight the importance of using simple tools, such as HRQoL assessments, during clinic visits to identify and address physical, mental, and emotional burdens, which could potentially improve overall well-being [34].

The perception of stigma among patients with T1DM can significantly hinder their utilization of healthcare services [35]. Our study is the first to assess stigma awareness among pediatric T1DM patients in Jordan, an issue that has received limited attention in this context. We found a stigma score of 2.64, which is lower than the 3.4 reported in a previous study in Turkey [36]. Additionally, we observed a negative association between stigma scores and BMI, which is inconsistent with findings by Liu et al. [37]. Furthermore, a lower level of stigma was associated with a family history of DM, contrasting with the findings of Celebi et al. [36]. These discrepancies may be attributed to differences in cultural and social perceptions, as well as varying levels of awareness about the disease within the population. Addressing stigma related to T1DM requires urgent action, including awareness campaigns, support groups, and strengthening household and specialized support structures.

Most notably, our study revealed a high prevalence of uncontrolled DM, which is consistent with previous findings in Jordan [15,16,18]. This highlights the need for comprehensive intervention that address all aspects of the disease, with a primary focus on improving adherence. While our patients showed slightly above-average adherence scores, the majority still exhibited poorer glycemic control. In line with previous research, we found that children with university-educated mothers and those with shorter durations of DM experienced better glycemic control, as reflected by lower HbA1c levels [17,38]. These findings underscore the critical role of education and early intervention in the effective management of T1DM.

The current study revealed that the majority of patients (94%) experienced hypoglycemic episodes in the previous 6 months, a prevalence higher than that reported in a prior study in Jordan (66%) [16]. Interestingly, our findings, along with those of Alkhatatbeh et al., showed similarities in the most commonly reported symptoms of hypoglycemia, with dizziness, tremors, and hunger being the most frequent symptoms in our cohort [16]. In this study, the occurrence of hypoglycemia was associated with BMI level, a finding supported by Zhang et al. [39]. This underscores the importance of increasing patients and guardians’ awareness of hypoglycemia symptoms, as this knowledge can enhance adherence, glycemic control, and overall quality of life. Additionally, within the previous 6 months, 28% of our patients experienced DKA, a higher incidence compared to a study by Kumar et al. (6.2%) [14]. In our study, inadequate or missed doses of insulin were identified as the primary cause of DKA, consistent with findings by Al- Hayek et al. [40]. Contrary to Kumar et al.’s findings, we observed that a higher incidence of DKA was associated with lower levels of HRQoL. Implementing protective measures against DKA, such as patient education and improved healthcare access, could improve HRQoL and reduce the strain on healthcare resources by decreasing DKA-related hospital admissions [14].

It is important to recognize that variations in study design, population characteristics, and sample size contribute to both the similarities and discrepancies between our findings and those of previous studies. While our study provides valuable insights, several limitations should be considered. First, the cross-sectional nature of the study limits the ability to establish causal relationships between the various factors analyzed. Second, there is a possibility of recall bias in participants’ responses, which could affect the accuracy of the data, particularly regarding hypoglycemia episodes, as these were assessed solely based on patient-reported symptoms and frequency. However, consistent data collection methods were used to mitigate this risk. Third, we were unable to use the zBMI score due to the lack of population-based BMI data in Jordan. Instead, we utilized conventional BMI, which has been applied in other recent studies involving children. Lastly, the presence of guardians during data collection may have influenced the participants’ responses. These limitations underscore the need for future longitudinal studies to better understand the relationships between factors affecting pediatric T1DM outcomes.

## Conclusions

The current study provides valuable insights into patient reported outcomes among pediatric patients with T1DM in Jordan. Our findings indicate that patients demonstrated better adherence to pharmaceutical management, particularly insulin, compared to adherence to physical and dietary therapies. Despite the majority of patients exhibiting poorly controlled disease and experiencing hypoglycemic episodes in the past 6 months, they reported above-average HRQoL. These results underscore the critical importance of patient education regarding their condition, adherence to treatment regimens, personalized diet plans, and physical activity. Additionally, the implementation of support campaigns is essential. Clinical pharmacists and dieticians can play a key role in delivering these services to optimize patient healthcare outcomes. Furthermore, we recommend future studies using cohort or interventional designs with larger sample sizes to enhance the generalizability of our findings and further explore the complex dynamics influencing pediatric T1DM management in Jordan. By continuing to investigate and address these factors, we can work toward improving the overall well-being and health outcomes of pediatric T1DM patients in our community.

## Supporting information

S1 Table Univariate and multivariate analyses of factors associated with diabetes related stigma. (DOCX)

S2 Table Univariate and multivariate analyses of factors affecting glycemic control (HbA1c). (DOCX)

S3 Table Features of hypoglycemia and DKA episodes among our patients. (DOCX)

S4 Table Univariate and multivariate analyses of factors associated with hypoglycemia incidence. (DOCX)

S5 Table Univariate and multivariate analyses of factors associated with DKA occurrence. (DOCX)

S1 File Study questionnaires. (PDF)

S2 File Raw data. (XLSX)
