# Supplementary material for: Type 1 diabetes mellitus in children: Patient reported outcomes
Source: PLoS One. 2025 May 5;20(5):e0322882. doi: 10.1371/journal.pone.0322882 (PMC12052175; doi:10.1371/journal.pone.0322882)
Supplement: S1 Table — (DOCX) [file pone.0322882.s001.docx]

**S1 Table.** **Univariate and multivariate analyses of factors associated with diabetes related stigma.**

| **Characteristics** | **Univariate analysis**  **N=150** | | **Multivariate analysis ^e^**  **N=150** | |
| --- | --- | --- | --- | --- |
|  | **Participants** | **P-value** | **Β** | **P-value** |
|  |  |  |  |  |
| Gender ^a^   - Male - Female | 2.65±0.32  2.62±0.35 | 0.589 |  |  |
| Patient’s age group ^a^   - < 12 years - ≥ 12 years | 2.67±0.32  2.59±0.36 | 0.165 |  |  |
| BMI ^b^ | -0.182 | 0.026 | - 0.016 | 0.041 |
| Mother’s level of education ^a^   - Basic education - University education | 2.6±0.36  2.69±0.29 | 0.097 | 0.068 | 0.224 |
| Family’s monthly income ^a^   - < 500 JD - ≥ 500 JD | 2.6±0.37  2.67±0.31 | 0.219 |  |  |
| Residence ^a^   - Urban areas - Rural areas | 2.67±0.35  2.57±0.29 | 0.117 |  |  |
| Family history for DM ^a^   - No - Yes | 2.7±0.31  2.6±0.35 | 0.063 | - 0.109 | 0.047 |
| Age at diagnosis (years) ^b^ | -0.046 | 0.573 |  |  |
| Duration of diabetes (years) ^c^ | -0.063 | 0.441 |  |  |
| HbA1c ^a^   - < 7% - ≥ 7% | 2.61±0.29  2.65±0.35 | 0.547 |  |  |
| Blood glucose level ^a^   - ≤ 130 mg/dl - > 130 mg/dl | 2.62±0.35  2.66±0.32 | 0.464 |  |  |
| Comorbidities ^a^   - No - Yes | 2.64±0.32  2.63±0.39 | 0.861 |  |  |
| Number of hypoglycemia episodes in the previous 6 months ^c^ | 0.046 | 0.578 |  |  |
| DKA occurrence in the previous 6 months ^a^   - No - Yes | 2.62±0.33  2.67±0.35 | 0.445 |  |  |
| Disease related factors ^a,d^   - No - Yes | 2.66±0.29  2.62±0.36 | 0.534 |  |  |
| Adherence score ^b^ | -0.027 | 0.744 |  |  |
| HRQoL score ^b^ | -0.018 | 0.826 |  |  |

Abbreviations: BMI, Body Mass Index; HbA1c, Glycated hemoglobin; HRQoL, Health Related-Quality of Life; DKA, Diabetic Ketoacidosis.

^a^ Data was described as mean ±SD and analyzed by independent t-test

^b^ Data was described as correlation coefficient and analyzed by Pearson correlation

^c^ Data was described as correlation coefficient and analyzed by Spearman correlation

^d^ Include school absent, emergency room visits, hospital admission

^e^ Multivariate analysis: linear regression
