# Supplementary material for: Type 1 diabetes mellitus in children: Patient reported outcomes
Source: PLoS One. 2025 May 5;20(5):e0322882. doi: 10.1371/journal.pone.0322882 (PMC12052175; doi:10.1371/journal.pone.0322882)
Supplement: S2 Table — (DOCX) [file pone.0322882.s002.docx]

**S2 Table.** **Univariate and multivariate analyses of factors affecting glycemic control (HbA1c).**

| **Characteristics ^a^** | **Univariate analysis**  **N=150** | | | **Multivariate analysis ^e^**  **N=150** | |
| --- | --- | --- | --- | --- | --- |
|  | **Controlled DM**  **(n=36)** | **Uncontrolled DM**  **(n=114)** | **P-value** | **OR (95% CI)** | **P-value** |
| Gender   - Male - Female | 19 (25.3%)  17(22.7%) | 56 (74.7%)  58 (77.3%) | 0.702 |  |  |
| Patient’s age group   - < 12 years - ≥ 12 years | 22 (25%)  14 (22.6%) | 66 (75%)  48 (77.4%) | 0.733 |  |  |
| BMI ^b^ | 18.22±3.97 | 18.8±3.47 | 0.402 |  |  |
| Family monthly income   - < 500 JD - ≥ 500 JD | 11 (18%)  25 (28.1%) | 50 (82%)  64 (71.9%) | 0.157 |  |  |
| Mother’s level of education   - Basic education - University education | 14(15.4%)  22(37.3%) | 77(84.6%)  37(62.7%) | 0.002 | 0.325(0.133-0.793) | 0.013 |
| Residency   - Urban areas - Rural areas | 30 (28%)  6 (14%) | 77 (72%)  37 (86%) | 0.068 | 2.041(0.72-5.799) | 0.18 |
| Family history of DM   - No - Yes | 15 (24.6%)  21 (23.6%) | 46 (75.4%)  68 (76.4%) | 0.889 |  |  |
| Duration of diabetes (years) ^c^ | - 1. [0.7-3] | 3 [1-5.5] | 0.011 | 1.272 (1.04-1.557) | 0.019 |
| Age at diagnosis (years) ^b^ | 7.39±4 | 7.23±3.5 | 0.817 |  |  |
| Blood glucose level   - ≤ 130 mg/dl - > 130 mg/dl | 22 (30.6%)  14 (17.9%) | 50 (69.4%)  64 (82.1%) | 0.071 | 1.426 (0.613-3.316) | 0.41 |
| Comorbidities   - No - Yes | 33 (26.4%)  3 (12%) | 92 (73.6%)  22 (88%) | 0.124 |  |  |
| Number of hypoglycemia episodes in the previous 6 months ^c^ | 12 [5-37.25] | 12 [5-31.25] | 0.898 |  |  |
| DKA occurrence in the previous 6 months   - No - Yes | 28 (25.9%)  8 (19%) | 80 (74.1%)  34 (81%) | 0.376 |  |  |
| Disease related factors ^a,d^   - No - Yes | 17 (28.8%)  19 (20.9%) | 42 (71.2%)  72 (79.1%) | 0.266 |  |  |
| Adherence score ^b^ | 63.47±19.01 | 55.48±17.5 | 0.021 | 0.996(0.971-1.022) | 0.756 |
| HRQoL score ^b^ | 67.81±12.79 | 61.83±11.23 | 0.008 | 0.966(0.927-1.008) | 0.11 |
| Stigma score ^b^ | 2.61±0.29 | 2.65±0.35 | 0.547 |  |  |

Abbreviations: BMI, Body Mass Index; HbA1c, Glycated hemoglobin; HRQoL, Health Related-Quality of Life; DKA, diabetic Ketoacidosis; DM, Diabetes Mellitus; OR, Odds Ratio; CI, Confidence Interval.

^a^ All data was expressed as n (%) of participants unless otherwise indicated and were analyzed by chi-square test

^b^ Data was described as mean ±SD and analyzed by independent t-test

^c^ Data was described as median [Interquartile range] and analyzed by Mann Whitney test

^d^ Include school absent, emergency room visits and hospital admission

^e^ Multivariate analysis: logistic regression
