# Supplementary material for: Type 1 diabetes mellitus in children: Patient reported outcomes
Source: PLoS One. 2025 May 5;20(5):e0322882. doi: 10.1371/journal.pone.0322882 (PMC12052175; doi:10.1371/journal.pone.0322882)
Supplement: S3 Table — (DOCX) [file pone.0322882.s003.docx]

**S3 Table. Features of hypoglycemia and DKA episodes among our patients.**

| **Measures** | **Values** |
| --- | --- |
| **Hypoglycemia** | |
| Hypoglycemia occurrence in the previous 6 months ^a^   - No - Yes | 9 (6%)  141 (94%) |
| Number of hypoglycemia episodes in the previous 6 months ^b^ | 12 [5-32.75] |
| Autonomic symptoms ^a^   - Shaking - Hunger - Sweating - Palpitation - Irritability - Anxiety | 62 (41.3%)  41 (27.3%)  34 (22.7%)  15 (10%)  10 (6.7%)  4 (2.7%) |
| Neuroglycopenic symptoms ^a^   - Dizziness - Fatigue - Headache - Feeling cold - Others | 85 (56.7%)  16 (10.7%)  9 (6%)  5 (3.3%)  15 (10%) |
| **Diabetic Ketoacidosis (DKA)** |  |
| DKA occurrence in the previous 6 months ^a^   - No - Yes | 108 (72%)  42 (28%) |
| Causes for DKA ^a^   - Missed or insufficient insulin doses - First-time diagnosis - Infection - Unknown | 12 (28.6%)  11 (26.2%)  10 (23.8%)  9 (21.4%) |

^a^ Data was expressed as n (%) of participants

^b^ Data were described as median [Interquartile range]
