# Supplementary material for: Type 1 diabetes mellitus in children: Patient reported outcomes
Source: PLoS One. 2025 May 5;20(5):e0322882. doi: 10.1371/journal.pone.0322882 (PMC12052175; doi:10.1371/journal.pone.0322882)
Supplement: S4 Table — (DOCX) [file pone.0322882.s004.docx]

**S4 Table. Univariate and multivariate analyses of factors associated with hypoglycemia incidence.**

| **Characteristics** | **Univariate analysis**  **N=150** | | **Multivariate analysis ^d^**  **N=150** | |
| --- | --- | --- | --- | --- |
|  | **Participants** | **P-value** | **Β** | **P-value** |
| Gender ^a^   - Male - Female | 15 [6-48]  12 [4-30] | 0.034 | 0.208 | 0.302 |
| Patient’s age group ^a^   - < 12 years - ≥ 12 years | 13.5 [6-35.75]  12 [3-30] | 0.117 |  |  |
| BMI ^b^ | - 0.218 | 0.007 | - 0.084 | 0.003 |
| Mother’s level of education ^a^   - Basic education - University education | 12 [4-30]  15 [6-36] | 0.177 |  |  |
| Family’s monthly income ^a^   - < 500 JD - ≥ 500 JD | 12 [2.5-32.5]  12 [6-34] | 0.178 |  |  |
| Residence ^a^   - Urban areas - Rural areas | 12 [5-36]  15 [6-30] | 0.501 |  |  |
| Family history for DM ^a^   - No - Yes | 12 [5-42]  12 [5-30] | 0.918 |  |  |
| Age at diagnosis (years) ^b^ | -0.12 | 0.142 |  |  |
| Duration of DM (years) ^b^ | 0.076 | 0.356 |  |  |
| HbA1c ^a^   - < 7% - ≥ 7% | 12 [5-37.25]  12 [5-31.25] | 0.898 |  |  |
| Blood glucose level ^a^   - ≤ 130 mg/dl - > 130 mg/dl | 13.5 [6-30]  12 [5-36] | 0.882 |  |  |
| Comorbidities ^a^   - No - Yes | 12 [6-33.5]  12 [3.5-30] | 0.343 |  |  |
| DKA occurrence in the previous 6 months ^a^   - No - Yes | 12 [5-36]  12 [5-25] | 0.9 |  |  |
| Disease related factors ^a,c^   - No - Yes | 15 [6-36]  12 [5-32] | 0.197 |  |  |
| Adherence score ^b^ | 0.126 | 0.125 |  |  |
| HRQoL score ^b^ | -0.078 | 0.346 |  |  |
| Stigma score ^b^ | 0.046 | 0.578 |  |  |

Abbreviations: BMI, Body Mass Index; HbA1c, Glycated hemoglobin; HRQoL, Health Related-Quality of Life; DKA, Diabetic Ketoacidosis.

^a^ Data was described as median [Interquartile range] and analyzed by Mann Whitney test

^b^ Data was described as correlation coefficient and analyzed by Spearman correlation

^c^ Include school absent, emergency room visits and hospital admission

^d^ Multivariate analysis: linear regression
