# Supplementary material for: Type 1 diabetes mellitus in children: Patient reported outcomes
Source: PLoS One. 2025 May 5;20(5):e0322882. doi: 10.1371/journal.pone.0322882 (PMC12052175; doi:10.1371/journal.pone.0322882)
Supplement: S5 Table — (DOCX) [file pone.0322882.s005.docx]

**S5 Table.** **Univariate and multivariate analyses of factors associated with DKA occurrence.**

| **Characteristics ^a^** | **Univariate analysis**  **N=150** | | | **Multivariate analysis ^d^**  **N=150** | |
| --- | --- | --- | --- | --- | --- |
|  | **No DKA (N=108)** | **DKA**  **(N=42)** | **P-value** | **OR (95% CI)** | **P-value** |
| Gender   - Male - Female | 58 (77.3%)  50 (66.7%) | 17 (22.7%)  25 (33.3%) | 0.146 |  |  |
| Patient’s age group   - < 12 years - ≥ 12 years | 61 (69.3%)  47 (75.8%) | 27 (30.7%)  15 (24.2%) | 0.383 |  |  |
| BMI ^b^ | 18.85± 3.82 | 18.16± 2.9 | 0.293 |  |  |
| Mother’s level of education   - Basic education - University education | 66 (72.5%)  42 (71.2%) | 25 (27.5%)  17 (28.8%) | 0.858 |  |  |
| Family’s monthly income   - < 500 JD - ≥ 500 JD | 40 (65.6%)  68 (76.4%) | 21 (34.4%)  21 (23.6%) | 0.147 |  |  |
| Family history for DM   - No - Yes | 42 (68.9%)  66 (74.2%) | 19 (31.1%)  23 (25.8%) | 0.477 |  |  |
| Residency   - Urban areas - Rural areas | 81 (75.7%)  27 (62.8%) | 26 (24.3%)  16 (37.2%) | 0.111 |  |  |
| HbA1c   - < 7% - ≥ 7% | 28 (77.8%)  80 (70.2%) | 8 (22.2%)  34 (29.8%) | 0.376 |  |  |
| Blood glucose level   - ≤ 130 mg/dl - > 130 mg/dl | 51 (70.8%)  57 (73.1%) | 21 (29.2%)  21 (26.9%) | 0.76 |  |  |
| Comorbidities   - No - Yes | 93 (74.4%)  15 (60%) | 32 (25.6%)  10 (40%) | 0.143 |  |  |
| Number of hypoglycemia episodes in the previous 6 months ^c^ | 12 [5-36] | 12 [5-25] | 0.9 |  |  |
| Adherence score ^b^ | 58.1±18.58 | 55.59±17.01 | 0.449 |  |  |
| HRQoL score ^b^ | 64.81±11.37 | 59.29±12.31 | 0.01 | 0.962 (0.933-0.992) | 0.012 |
| Stigma score ^b^ | 2.62±0.33 | 2.67±0.35 | 0.445 |  |  |

Abbreviations: BMI, Body Mass Index; HbA1c, Glycated hemoglobin; HRQoL, Health Related-Quality of Life; DKA, diabetic Ketoacidosis; OR, Odds Ration; CI, Confidence Interval.

^a^ All data was expressed as n (%) of participants unless otherwise indicated and were analyzed by chi-square test

^b^ Data was described as mean ±SD and analyzed by independent t-test

^c^ Data was described as median [Interquartile range] and analyzed by Mann Whitney test

^d^ Multivariate analysis: logistic regression
