# Supplementary material for: Type 1 diabetes mellitus in children: Patient reported outcomes
Source: PLoS One. 2025 May 5;20(5):e0322882. doi: 10.1371/journal.pone.0322882 (PMC12052175; doi:10.1371/journal.pone.0322882)
Supplement: S1 File — (PDF) [file pone.0322882.s006.pdf]

DIABETES MANAGEMENT QUESTIONNAIRE (DMQ)[1]

Diabetes management involves many different things. Families may do some of these things more often than others. The questions below are about how you and your parents/guardians have taken care of your diabetes in the past month. For each question, choose the answer that best describes how often that item happened during the PAST MONTH.

Think about your PHYSICAL ACTIVITY (sports, active play, etc.) during the PAST MONTH.

How often did you or your parent/guardian…	Almost Never	Some-times	Half of the time	Most of the time	Almost Always
1. Adjust your food or insulin before long periods of physical activity?	①	②	③	④	⑤
2. Check your blood sugar before physical activity?	①	②	③	④	⑤
3. Check your blood sugar within 2 to 3 hours after physical activity?	①	②	③	④	⑤

Think about your MEAL AND SNACK TIMES during the PAST MONTH.

How often did you or your parent/guardian…	Almost Never	Some-times	Half of the time	Most of the time	Almost Always
4. Use a blood sugar result to help decide the amount of insulin to give for a meal?	①	②	③	④	⑤
5. Use the amount of carbohydrate to help decide the amount of insulin to give for a meal?	①	②	③	④	⑤
6. Use food labels to help count carbohydrates (when food labels were available)?	①	②	③	④	⑤
7. Measure or weigh food to help count carbohydrates, when eating at home?	①	②	③	④	⑤
8. Give all of an insulin dose after you finished eating?	①	②	③	④	⑤
9. Eat meals without knowing the amount of carbohydrate?	①	②	③	④	⑤
10. Eat meals without checking a blood sugar?	①	②	③	④	⑤
11. Check your blood sugar within 2 hours after a meal?	①	②	③	④	⑤

Think about LOW BLOOD SUGARS during the PAST MONTH.

How often did you or your parent/guardian…	Almost Never	Some-times	Half of the time	Most of the time	Almost Always
12. Check your blood sugar before treating a low blood sugar?	①	②	③	④	⑤
13. Check your blood sugar after treating a low blood sugar?	①	②	③	④	⑤
14. Over-treat a low blood sugar with more carbohydrates than were needed?	①	②	③	④	⑤

(continued on next page)

Think about HIGH BLOOD SUGARS during the PAST MONTH.

How often did you or your parent/guardian…	Almost Never	Some-times	Half of the time	Most of the time	Almost Always
15. Give a dose of insulin right away based on a correction/sensitivity factor or sliding scale?	①	②	③	④	⑤
16. Re-check your blood sugar about 2 hours after giving insulin for a high blood sugar?	①	②	③	④	⑤
17. Check ketones when you had 2 blood sugar results in a row above 250-300? ☐ *Not applicable*	①	②	③	④	⑤

Think about INSULIN and CHECKING BLOOD SUGARS during the PAST MONTH.

How often did you or your parent/guardian…	Almost Never	Some-times	Half of the time	Most of the time	Almost Always
18. Go more than 8 hours without checking your blood sugar, except for overnight?	①	②	③	④	⑤
19. Check your blood sugar at bedtime?	①	②	③	④	⑤
20. Miss or forget an insulin dose?	①	②	③	④	⑤

Ref.1: Mehta SN, Nansel TR, Volkening LK, Butler DA, Haynie DL, Laffel LM. Validation of a contemporary adherence measure for children with Type 1 diabetes: the Diabetes Management Questionnaire. Diabetic medicine : a journal of the British Diabetic Association. 2015;32(9):1232-8. Epub 2015/08/19. doi: 10.1111/dme.12682.

Quality of life[1,2]
CHILD REPORT

DIRECTIONS

Children with diabetes sometimes have special problems. Please tell us **how much of a problem** each one has been for you during the **past ONE month** by circling:

- **0** if it is **never** a problem
- **1** if it is **almost never** a problem
- **2** if it is **sometimes** a problem
- **3** if it is **often** a problem
- **4** if it is **almost always** a problem

There are no right or wrong answers.

*In the past **ONE month,** how much of a **problem** has this been for you*

ABOUT MY DIABETES *(problems with…)*	Never	Almost Never	Sometimes	Often	Almost Always
**1. I feel hungry**	0	1	2	3	4
**2. I feel thirsty**	0	1	2	3	4
**3. I have to go to the bathroom too often**	0	1	2	3	4
**4. I have stomachaches**	0	1	2	3	4
**5. I have headaches**	0	1	2	3	4
**6. I go "low"**	0	1	2	3	4
**7. I feel tired or fatigued**	0	1	2	3	4
**8. I get shaky**	0	1	2	3	4
**9. I get sweaty**	0	1	2	3	4
**10. I have trouble sleeping**	0	1	2	3	4
**11. I get irritable**	0	1	2	3	4

TREATMENT - I *(problems with…)*	Never	Almost Never	Sometimes	Often	Almost Always
**1. It hurts to prick my finger or give insulin shots**	0	1	2	3	4
**2. I am embarrassed about having diabetes**	0	1	2	3	4
**3. My parents and I argue about my diabetes care**	0	1	2	3	4
**4. It is hard for me to stick to my diabetes care plan**	0	1	2	3	4

*Whether you do these things **on your own or with the help of your parents**, please answer how hard these things were to do in the past **ONE month**.*

TREATMENT II - *(problems with…)*	Never	Almost Never	Sometimes	Often	Almost Always
**1. It is hard for me to take blood glucose tests**	0	1	2	3	4
**2. It is hard for me to take insulin shots**	0	1	2	3	4
**3. It is hard for me to exercise**	0	1	2	3	4
**4. It is hard for me to keep track of carbohydrates or exchanges**	0	1	2	3	4

**5. It is hard for me to wear my id bracelet**	0	1	2	3	4
**6. It is hard for me to carry a fast-acting carbohydrate**	0	1	2	3	4
**7. It is hard for me to eat snacks**	0	1	2	3	4

WORRY *(problems with…)*	Never	Almost Never	Sometimes	Often	Almost Always
**1. I worry about "going low"**	0	1	2	3	4
**2. I worry about whether or not my medical treatments are working**	0	1	2	3	4
**3. I worry about long-term complications from diabetes**	0	1	2	3	4

In the past ONE month, how much of a problem has this been for you …

**COMMUNICATION *(problems with…)***	Never	Almost Never	Sometimes	Often	Almost Always
**1. It is hard for me to tell the doctors and nurses how I feel**	0	1	2	3	4
**2. It is hard for me to ask the doctors and nurses questions**	0	1	2	3	4
**3. It is hard for me to explain my illness to other people**	0	1	2	3	4

Quality of life
PARENT REPORT

DIRECTIONS

Children with diabetes sometimes have special problems. On the following page is a list of things that might be a problem for **your child**. Please tell us **how much of a problem** each one has been for **your child** during the **past ONE month** by circling:

- **0** if it is **never** a problem
- **1** if it is **almost never** a problem
- **2** if it is **sometimes** a problem
- **3** if it is **often** a problem
- **4** if it is **almost always** a problem

There are no right or wrong answers.
If you do not understand a question, please ask for help.

*In the past **ONE month,** how much of a **problem** has your child had with …*

DIABETES *(problems with…)*	Never	Almost Never	Sometimes	Often	Almost Always
**1. Feeling hungry**	0	1	2	3	4
**2. Feeling thirsty**	0	1	2	3	4
**3. Having to go to the bathroom too often**	0	1	2	3	4
**4. Having stomachaches**	0	1	2	3	4
**5. Having headaches**	0	1	2	3	4
**6. Going "low"**	0	1	2	3	4
**7. Feeling tired or fatigued**	0	1	2	3	4
**8. Getting shaky**	0	1	2	3	4
**9. Getting sweaty**	0	1	2	3	4
**10. Having trouble sleeping**	0	1	2	3	4
**11. Getting irritable**	0	1	2	3	4

TREATMENT - I *(problems with…)*	Never	Almost Never	Sometimes	Often	Almost Always
**1. Needle sticks (i.e. injections/blood tests) causing him/her pain**	0	1	2	3	4
**2. Getting embarrassed about having diabetes**	0	1	2	3	4
**3. Arguing with me or my spouse about diabetes care**	0	1	2	3	4
**4. Sticking to his/her diabetes care plan**	0	1	2	3	4

*Whether your child does these things **independently or with your help**, please answer how difficult these things were to do in the past **ONE month**. (Note: This section is **not** asking about your child's independence in these areas, just how hard they were to do).*

TREATMENT - II (*problems with…)*	Never	Almost Never	Sometimes	Often	Almost Always
**1. It is hard for my child to take blood glucose tests**	0	1	2	3	4
**2. It is hard for my child to take insulin shots**	0	1	2	3	4
**3. It is hard for my child to exercise**	0	1	2	3	4
**4. It is hard for my child to track carbohydrates or exchanges**	0	1	2	3	4
**5. It is hard for my child to wear his/her id bracelet**	0	1	2	3	4
**6. It is hard for my child to carry a fast-acting carbohydrate**	0	1	2	3	4
**7. It is hard for my child to eat snacks**	0	1	2	3	4

WORRY *(problems with…)*	Never	Almost Never	Sometimes	Often	Almost Always
**1. Worrying about "going low"**	0	1	2	3	4
**2. Worrying about whether or not medical treatments are working**	0	1	2	3	4
**3. Worrying about long-term complications of diabetes**	0	1	2	3	4

In the past ONE month, how much of a problem has your child had with…

**COMMUNICATION *(problems with…)***	Never	Almost Never	Sometimes	Often	Almost Always
**1. Telling the doctors and nurses how he/she feels**	0	1	2	3	4
**2. Asking the doctors and nurses questions**	0	1	2	3	4
**3. Explaining his/her illness to other people**	0	1	2	3	4

Ref.1. Varni JW, Burwinkle TM, Jacobs JR, Gottschalk M, Kaufman F, Jones KL. The PedsQL in type 1 and type 2 diabetes: reliability and validity of the Pediatric Quality of Life Inventory Generic Core Scales and type 1 Diabetes Module. Diabetes Care. 2003;26(3):631-7.
Ref.2. Abdul-Rasoul M, Alotaibi F, Almahdi M, Alkandari H. Reliability and validity of the Arabic version of the PedsQL TM 4.0 generic ore scales and PedsQL TM 3.0 diabetes module. Journal of Diabetes Mellitus. 2012;02. doi: 10.4236/jdm.2012.23047.

Child Attitude Toward Illness Scale (CATIS)[1,2]

	1	2	3	4	5
**1- How good or bad do you feel it is that you have asthma?***	Very good	A little good	Not sure	A little bad	Very bad
**2- How fair is it that you have asthma?***	Very fair	A little fair	Not sure	A little unfair	Very unfair
**3- How happy or sad is it for you to have asthma?**	Very sad	A little sad	Not sure	A little happy	Very happy
**4- How embarrassed are you by telling others that you have asthma?***	Never	Not often	Not sure	Slightly embarrassed	Very embarrassed
**5- How often do you feel that your asthma is your fault?***	Never	Not often	Sometimes	Often	Very often
**6-How often do you feel that your asthma keeps you from doing thing you like to do?**	Very often	Often	Sometimes	Not often	Never
**7-How often do you feel that you will always be sick?***	Never	Not often	Sometimes	Often	Very often
**8-How often do you feel that your asthma keeps you from starting new things?**	Very often	Often	Sometimes	Not often	Never
**9-How often do you feel different from others because of your asthma?***	Never	Not often	Sometimes	Often	Very often
**10- How often do you feel bad because you have asthma?**	Very often	Often	Sometimes	Not often	Never
**11-How often do you feel sad about being sick?***	Never	Not often	Sometimes	Often	Very often

**12- How often do you feel embarrassed to use asthma medications in front of others (friends and relatives)?**	Very often	Often	Sometimes	Not often	Never
**13-How often do you feel just as good as other kids your age even though you have asthma?***	Very often	Often	Sometimes	Not often	Never

* Reversed for coding

Ref.1: Austin JK, Huberty TJ. Development of the Child Attitude Toward Illness Scale. Journal of pediatric psychology. 1993; 18(4):467-80.
Ref.2: Almomani BA, Al-Qawasmeh BS, Al-Shatnawi SF, Awad S, Alzoubi SA. Predictors of proper inhaler technique and asthma control in pediatric patients with asthma. Pediatric pulmonology. 2021;56(5):866-74. Epub 2021/01/27. doi: 10.1002/ppul.25263.
